# Preoperative Neutrophil to Lymphocyte Ratio and Platelet to Lymphocyte Ratio are Associated with the Prognosis of Group 3 and Group 4 Medulloblastoma

**DOI:** 10.1038/s41598-019-49733-6

**Published:** 2019-09-13

**Authors:** Ke Li, Wen-chao Duan, Hai-biao Zhao, Li Wang, Wei-wei Wang, Yun-bo Zhan, Tao Sun, Feng-jiang Zhang, Bin Yu, Ya-hui Bai, Yan-min Wang, Yu-chen Ji, Jin-qiao Zhou, Xian-zhi Liu, Zhi-feng Zhang, Zhen-yu Zhang

**Affiliations:** 1 Department of Neurosurgery, The First Affiliated Hospital of Zhengzhou University, Zhengzhou, Henan 450052 China; 2 Department of Pathology, The First Affiliated Hospital of Zhengzhou University, Zhengzhou, Henan 450052 China

**Keywords:** CNS cancer, Surgical oncology

## Abstract

Inflammation and immunoreaction markers were correlated with the survival of patients in many tumors. However, there were no reports investigating the relationships between preoperative hematological markers and the prognosis of medulloblastoma (MB) patients based on the molecular subgroups (WNT, SHH, Group 3, and Group 4). A total 144 MB patients were enrolled in the study. The differences of preoperative hematological markers among molecular subgroups of MB were compared by One-way ANOVA method. Kaplan-Meier method was used to calculate the curves of progression free survival (PFS) and overall survival (OS). The comparison of survival rates in different groups were conducted by the Log-rank test. Multivariate analysis was used to evaluate independent prognostic factors. Increased preoperative NLR (neutrophil-to-lymphocyte ratio, PFS, *P* = 0.004, OS, *P* < 0.001) and PLR (platelet-to-lymphocyte ratio, PFS, *P* = 0.028, OS, *P* = 0.003) predicted poor prognosis in patients with MB, while preoperative MLR (monocyte-to-lymphocyte ratio), MPV (mean platelet volume), PDW (platelet distribution width), and AGR (albumin-to-globulin ratio) were revealed no predictive value on the prognosis of patients with MB. Furthermore, high preoperative NLR and PLR predicted unfavorable prognosis in childhood MB patients. However, preoperative NLR and PLR were not associated with the prognosis in adult MB patients. Multivariate analysis demonstrated preoperative NLR (PFS, *P* = 0.029, OS, *P* = 0.005) and PLR (PFS, *P* = 0.023, OS, *P* = 0.005) were the independent prognostic factors in MB patients. Emphatically, the levels of preoperative NLR and PLR in Group 3 MB were significantly higher than those in WNT MB. High preoperative NLR was associated with unfavorable OS in Group 3 (*P* = 0.032) and Group 4 (*P* = 0.027) tumors. Similarly, increased preoperative PLR predicted poor PFS (*P* = 0.012) and OS (*P* = 0.009) in Group 4 tumors. Preoperative NLR and PLR were the potential prognostic markers for MB patients. Preoperative NLR and PLR were significantly associated with the survival of Group 3 and Group 4 tumors.

## Introduction

Medulloblastoma (MB) is one of the most common brain malignant tumors in children, which accounts approximately 20% of all the pediatric brain tumors^1^. Although the prognosis of patients with MB has been improved by the advancement radiation therapy and chemotherapy, the survival rates of patients with MB remain considerably different. Exploring new markers to accurately predict the prognosis of patients with MB could contribute to the evaluation and management of the disease.

Recently, more and more studies demonstrated that the preoperative hematological markers played important roles in predicting prognosis of several types of tumors, such as esophageal cancer, colorectal cancer, renal cell carcinoma, and glioblastoma, etc.^2–5^. Specifically, increased preoperative neutrophil-to-lymphocyte ratio (NLR), platelet-to-lymphocyte ratio (PLR), and monocyte-to-lymphocyte ratio (MLR) were reported poor prognosis in patients with solid cancers^6–8^. Reduced preoperative mean platelet volume (MPV) was discovered in patients with renal cell carcinoma compared to the patients with benign renal tumor^9^. Moreover, preoperative platelet distribution width (PDW) was demonstrated as an independent risk factor for the prognosis with gastric cancer^10^. However, to our knowledge, no studies have investigated the prognostic significance of preoperative hematological markers in MB patients.

The determination of molecular subgroups was one of the most important advancements in the realm of MB investigation. Mounting evidences demonstrated that MB is a heterogenous disease and composed of different molecular subgroups: sonic hedgehog (SHH), wingless (WNT), Group 3, and Group 4. These subgroups are significantly different in transcriptional profiles, genetic abnormalities, clinical characteristics, and prognosis^1,11^. Recently, the four MB molecular subgroups have been included in the newest 2016 World Health Organization classification of tumors of the central nervous system^12^. Nevertheless, whether the prognostic value of hematological markers differ in molecular subgroups of MB remains unexplored.

The purpose of the present study was to investigate the prognostic significance of the preoperative hematological markers (NLR, PLR, MLR, MPV, PDW, and albumin-to-globulin ratio (AGR)) combined with molecular subgroups (WNT, SHH, Group 3, and Group 4) on the survival of patients with MB.

## Results

### Clinicopathologic characteristics

144 MB patients had complete preoperative hematological markers without preoperative infection diseases and systemic comorbidities were enrolled in the study (Supplementary Fig. 1), including 25 WNT, 30 SHH, 51 Group 3, and 38 Group 4 MB. The mean preoperative NLR for WNT, SHH, Group 3 and Group 4 MB were 1.56 ± 0.84, 2.19 ± 1.81, 4.02 ± 3.77, and 3.31 ± 5.50. The mean preoperative PLR for WNT, SHH, Group 3 and Group 4 MB were 110.46 ± 51.28, 130.28 ± 74.98, 163.66 ± 74.47, 167.85 ± 138.77. The mean preoperative MLR, MPV, PDW, and AGR for 144 MB patients were 0.27 ± 0.45, 8.55 ± 1.42, 15.68 ± 1.42, 1.93 ± 0.44, respectively (Supplementary Table 1). Clinicopathologic data of the 116 MB patients were successfully followed up exhibited in Tables 1 and 2. 93 (80.2%) patients were children and 23 (19.8%) cases were adults. The mean age of the cohort was 12 years (ranged from 0.9 to 50 years). 75 (64.7%) patients were male and 41 (35.3%) were female. 79 (68.1%) patients’ preoperative karnofsky performance status scale (KPS) ≥ 80 and 37 (31.9%) patients’ preoperative KPS < 80. Furthermore, 88 (75.9%) tumors located in midline, and 28 (24.1%) tumors located in lateral. All the tumors underwent resection: 62 (53.4%) tumors had gross total resection, 54 (46.6%) had subtotal resection. 93 (80.2%) patients received postoperative primary radiotherapy (RT), 64 (55.2%) patients received postoperative primary chemotherapy (CHT).

**Table 1:** Univariate analysis of prognostic parameters for PFS in medulloblastoma patients (n = 116).

Parameters	No. of cases	5-year PFS (%)	*P*-value
Sex			
Male	75 (64.7%)	48	0.801
Female	41 (35.3%)	49.2	
Age			
Children	93 (80.2%)	43.3	0.109
Adults	23 (19.8%)	70.6	
PreKPS			
<80	37 (31.9%)	38.2	0.387
≥80	79 (68.1%)	52.1	
Location			
Midline	88 (75.9%)	39.1	**0.048**
Lateral	28 (24.1%)	70.4	
Extent of resection			
Gross Total	62 (53.4%)	71.0	**<0.001**
Subtotal	54 (46.6%)	24.4	
RT			
Yes	93 (80.2%)	52.4	**0.003**
No	23 (19.8%)	31.5	
CHT			
Yes	64 (55.2%)	46.9	0.564
No	52 (44.8%)	51.8	
NLR			
NLR ≤ 4.94	102 (87.9%)	51.3	**0.004**
NLR > 4.94	14 (12.1%)	28.6	
PLR			
PLR ≤ 142.31	72 (62.1%)	55.0	**0.028**
PLR > 142.31	44 (37.9%)	38.5	
MLR			
MLR ≤ 0.33	102 (87.9%)	49.8	0.157
MLR > 0.33	14 (12.1%)	39.2	
MPV			
MPV ≤ 8.80	88 (75.9%)	44.6	0.303
MPV > 8.80	28 (24.1%)	60.0	
PDW			
PDW ≤ 15.90	43 (37.1%)	37.8	0.096
PDW > 15.90	73 (62.9%)	56.0	
AGR			
AGR ≤ 1.59	22 (19.0%)	32.9	0.22
AGR > 1.59	94 (81.0%)	52.7	

PreKPS: preoperative karnofsky performance status scale.

RT: postoperative primary radiotherapy.

CHT: postoperative primary chemotherapy.

NLR: neutrophil-to-lymphocyte ratio.

PLR: platelet-to-lymphocyte ratio.

MLR: monocyte-to-lymphocyte ratio.

MPV: mean platelet volume.

PDW: platelet distribution width.

AGR: preoperative albumin-to-globulin ratio.

**Table 2:** Univariate analysis of prognostic parameters for OS in medulloblastoma patients (n = 116).

Parameters	No. of cases	5-year OS (%)	*P*-value
Sex			
Male	75 (64.7%)	52.2	0.242
Female	41 (35.3%)	69.9	
Age			
Children	93 (80.2%)	51.6	0.096
Adults	23 (19.8%)	79.1	
PreKPS			
<80	37 (31.9%)	54.6	0.938
≥80	79 (68.1%)	59.4	
Location			
Midline	88 (75.9%)	49.7	0.108
Lateral	28 (24.1%)	77.1	
Extent of resection			
Gross Total	62 (53.4%)	81.6	**<0.001**
Subtotal	54 (46.6%)	32.8	
RT			
Yes	93 (80.2%)	64.1	<**0.001**
No	23 (19.8%)	32.8	
CHT			
Yes	64 (55.2%)	56	0.469
No	52 (44.8%)	63.7	
NLR			
NLR ≤ 4.94	102 (87.9%)	62.5	<**0.001**
NLR > 4.94	14 (12.1%)	27.8	
PLR			
PLR ≤ 147.50	77 (66.4%)	69.9	**0.003**
PLR > 147.50	39 (33.6%)	38.8	
MLR			
MLR ≤ 0.12	12 (10.3%)	47.6	0.124
MLR > 0.12	104 (89.7%)	58.2	
MPV			
MPV ≤ 8.00	51 (44.0%)	46	0.625
MPV > 8.00	65 (56.0%)	64.9	
PDW			
PDW ≤ 15.90	43 (37.1%)	43.2	0.052
PDW > 15.90	73 (62.9%)	69.1	
AGR			
AGR ≤ 1.59	22 (19.0%)	38	0.053
AGR > 1.59	94 (81.0%)	63.5	

PreKPS: preoperative karnofsky performance status scale.

RT: postoperative primary radiotherapy.

CHT: postoperative primary chemotherapy.

NLR: neutrophil-to-lymphocyte ratio.

PLR: platelet-to-lymphocyte ratio.

MLR: monocyte-to-lymphocyte ratio.

MPV: mean platelet volume.

PDW: platelet distribution width.

AGR: preoperative albumin-to-globulin ratio.

### The comparison of preoperative hematological markers between WNT, SHH, Group 3, and Group 4 MB

**Figure 1:**
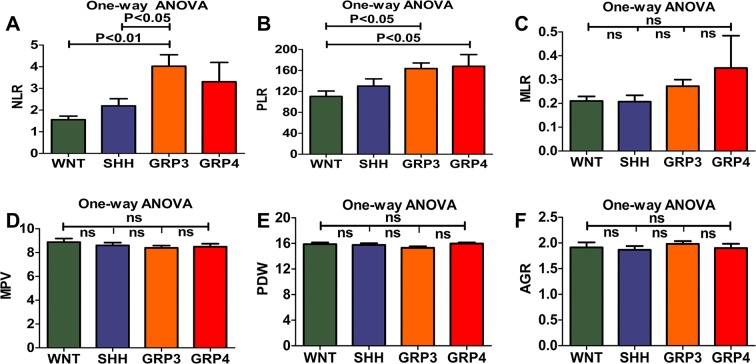
The levels of preoperative NLR, PLR, MLR, MPV, PDW and AGR presented among WNT, SHH, Group 3, and Group 4 were analyzed by One-way ANOVA. (**A**) The levels of preoperative NLR in Group 3 MB were significantly higher than those in WNT (P < 0.01) and SHH MB (P < 0.05). (**B**) The levels of preoperative PLR in Group 3 (P < 0.05) and Group 4 MB (P < 0.05) were higher than those in WNT MB. (**C**–**F**) The levels of preoperative MLR, MPV, PDW, and AGR among WNT, SHH, Group 3, and Group 4 had no statistically significant.

We compared the preoperative hematological markers among 144 MB patients by One-way ANOVA. It revealed that NLR (*P* < 0.01, Fig. 1A, *P* < 0.05, Fig. 1A) in Group 3 MB were significantly higher than those in WNT and SHH MB. The levels of PLR (*P* < 0.05, Fig. 1B, *P* < 0.05, Fig. 1B) in Group 3 and Group 4 MB were higher than those in WNT MB (Supplementary Table 1).

### Preoperative NLR and PLR were the independent prognostic markers in MB patients

**Figure 2:**
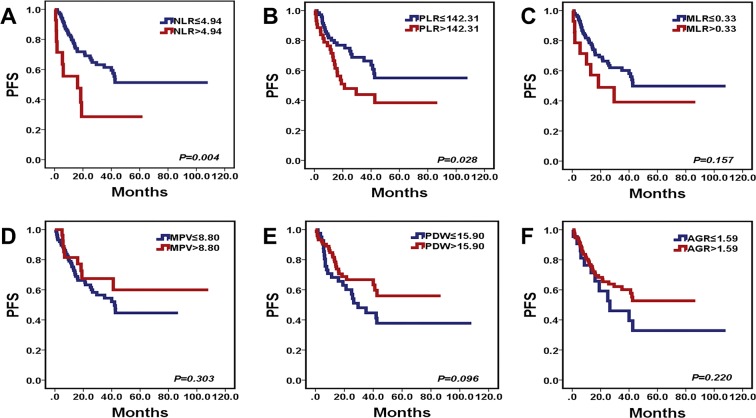
Kaplan-Meier survival curves for PFS probability according to preoperative NLR, PLR, MLR, MPV, PDW, and AGR levels. (**A**,**B**) High preoperative NLR (*P* = 0.004) and PLR (*P* = 0.028) predicted short PFS in patients with MB. (**C**–**F**) The levels of preoperative MLR, MPV, PDW, and AGR exited no statistically significant for PFS (*P* = 0.157, *P* = 0.303, *P* = 0.096, *P* = 0.220) in patients with MB.

**Figure 3:**
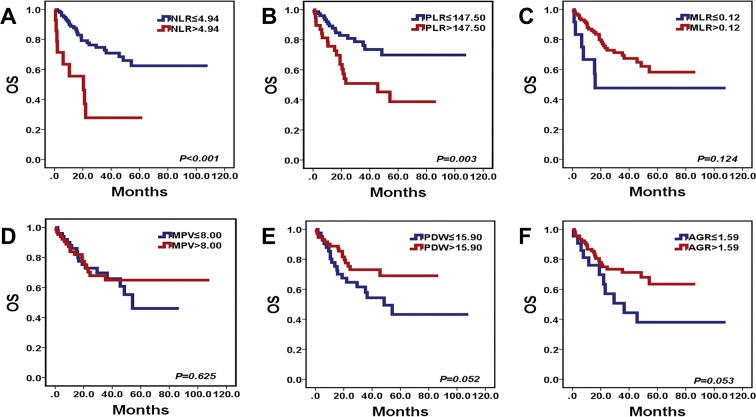
Kaplan-Meier survival curves for OS probability according to preoperative NLR, PLR, MLR, MPV, PDW and AGR levels. (**A**,**B**) High preoperative NLR and PLR predicted short OS (*P* < 0.001, *P* = 0.003) in patients with MB. (**C**–**F**) The levels of preoperative MLR, MPV, PDW, and AGR had no prognostic value for OS (*P* = 0.124, *P* = 0.625, *P* = 0.052, *P* = 0.053) in patients with MB.

We used the X-tile 3.6.1 software to calculate the cutoff values, and the cutoff values of the preoperative NLR, PLR, MLR, MPV, PDW and AGR for progression free survival (PFS) and overall survival (OS) were listed in Tables 1 and 2. Following 116 MB patients were analysed by univariate analysis (Supplementary Fig. 1). High preoperative NLR (*P* = 0.004, *P* < 0.001, Tables 1 and 2, Figs 2A and 3A) predicted unfavorable PFS and OS in MB patients. Similarly, high preoperative PLR (*P* = 0.028, *P* = 0.003, Tables 1 and 2, Figs 2B and 3B) was correlated with unfavorable PFS and OS in patients with MB. Subtotal resection (*P* < 0.001, *P* < 0.001, Tables 1 and 2) and no RT (*P* = 0.003, *P* < 0.001, Tables 1 and 2) were significantly associated with unfavorable PFS and OS with patients in MB. The tumors located in midline predicted poor PFS (*P* = 0.048, Table 1) in patients with MB.

In multivariate analysis, we analyzed preoperative NLR and PLR separately because they were strongly correlated and interfered with each other^13^. The results revealed that preoperative NLR (PFS, *P* = 0.029, OS, *P* = 0.005, Table 3), preoperative PLR (OS, *P* = 0.012, Table 4), the extent of resection (PFS, *P* = 0.012, OS, *P* = 0.011, Table 3, PFS, *P* = 0.014, OS, *P* = 0.014, Table 4) and RT (PFS, *P* = 0.009, OS, *P* = 0.001, Table 3, PFS, *P* = 0.010, OS, *P* = 0.001, Table 4) were the independent prognostic factors for the MB patients. When the factor of molecular subgroups was added in multivariate analyses, preoperative NLR lost independent significance in the multivariate analysis for PFS (*P* = 0.090, Supplementary Table 2). After adjusting for molecular subgroups, preoperative NLR and PLR were still the independent prognostic factors (OS, *P* = 0.013, Supplementary Table 2, OS, *P* = 0.014, Supplementary Table 3).

**Table 3:** Multivariate analysis of prognostic parameters for PFS and OS in medulloblastoma patients (n = 116).

Parameters	PFS		OS	
	OR (95%CI)	*P-*value	OR (95%CI)	*P-*value
Age				
Children	Reference		Reference	
Adults	0.965 (0.372–2.504)	0.942	0.994 (0.306–3.233)	0.992
Extent of resection				
Gross Total	Reference		Reference	
Subtotal	2.337 (1.201–4.546)	**0.012**	2.895 (1.269–6.601)	**0.011**
RT				
Yes	Reference		Reference	
No	2.415 (1.241–4.702)	**0.009**	3.420 (1.635–7.153)	**0.001**
Location				
Midline	Reference		Reference	
Lateral	0.668 (0.294–1.513)	0.333	0.760 (0.296–1.955)	0.570
NLR	1.046 (1.005–1.089)	**0.029**	1.061 (1.018–1.106)	**0.005**

OR: odds ratio.

CI: confidence interval.

RT: postoperative primary radiotherapy.

NLR: neutrophil-to-lymphocyte ratio.

**Table 4:** Multivariate analysis of prognostic parameters for PFS and OS in medulloblastoma patients (n = 116).

Parameters	PFS		OS	
	OR (95%CI)	*P-*value	OR (95%CI)	*P-*value
Age				
Children	Reference		Reference	
Adults	0.990 (0.389–2.615)	0.986	0.965 (0.327–3.462)	0.917
Extent of resection				
Gross Total	Reference		Reference	
Subtotal	2.313 (1.188–4.504)	**0.014**	2.817 (1.233–6.438)	**0.014**
RT				
Yes	Reference		Reference	
No	2.419 (1.240–4.719)	**0.010**	3.478 (1.657–7.300)	**0.001**
Location				
Midline	Reference		Reference	
Lateral	0.625 (0.276–1.413)	0.259	0.689 (0.269–1.762)	0.437
PLR	1.002 (0.997–1.004)	0.069	1.002 (1.001–1.004)	**0.012**

OR: odds ratio.

CI: confidence interval.

RT: postoperative primary radiotherapy.

PLR: platelet-to-lymphocyte ratio.

### Survival analysis of preoperative NLR and PLR in childhood and adult MB patients

**Figure 4:**
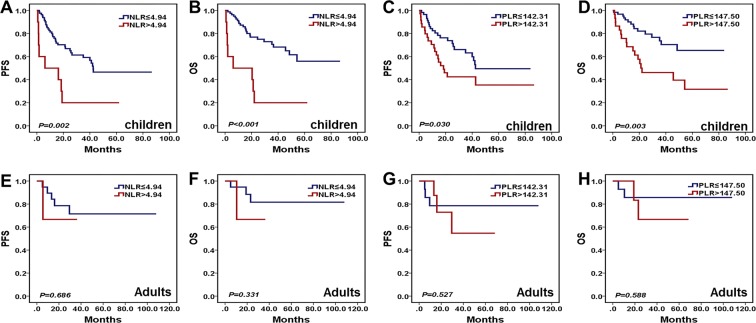
Kaplan-Meier survival curves for PFS and OS probability according to preoperative NLR and PLR in childhood and adult MB patients. (**A**–**D**) High preoperative NLR (PFS, *P* = 0.002, OS, *P* < 0.001) and PLR (PFS, *P* = 0.030, OS, *P* = 0.003) predicted worse prognosis in childhood MB patients. (**E**–**H**) the levels of preoperative NLR and PLR had no prognostic value for PFS (*P* = 0.686, *P* = 0.527) and OS (*P* = 0.331, *P* = 0.588) in adult MB patients.

Survival analysis was performed in 93 cases childhood MB patients and 23 cases adult MB patients. The results revealed that high preoperative NLR (PFS, *P* = 0.002, Fig. 4A, OS, *P* < 0.001, Fig. 4B) and PLR (PFS, *P* = 0.030, Fig. 4C, OS, *P* = 0.003, Fig. 4D) predicted worse prognosis in childhood MB patients. However, the levels of preoperative NLR and PLR had no prognostic value for PFS (*P* = 0.686, Fig. 4E, *P* = 0.527, Fig. 4G) and OS (*P* = 0.331, Fig. 4F, *P* = 0.588, Fig. 4H) in adult MB patients.

### Survival analysis of preoperative NLR and PLR in molecular subgroups

**Figure 5:**
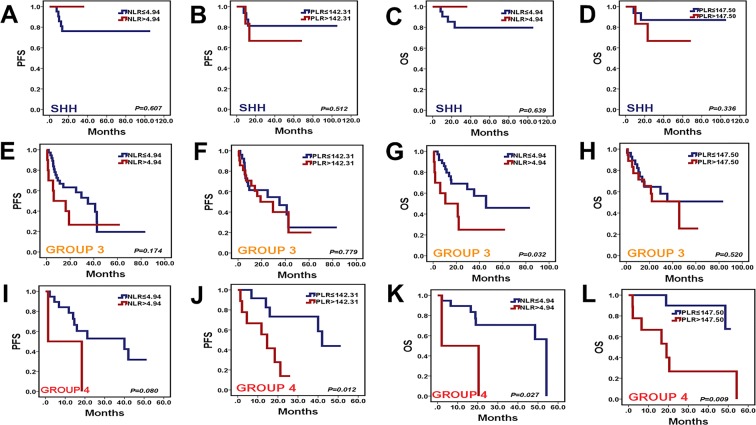
Kaplan-Meier survival curves for PFS and OS probability according to preoperative NLR and PLR levels in SHH, Group 3, and Group 4 MB. (**A**–**D**) The levels of preoperative NLR and PLR exited no statistically significant for PFS (*P* = 0.607, *P* = 0.512) and OS (*P* = 0.639, *P* = 0.336) in patients with SHH MB. (**E**,**F**) The levels of preoperative NLR and PLR had no prognostic value for PFS (*P* = 0.174, *P* = 0.779) in Group 3 MB. (**G**) High preoperative NLR predicted poor OS (*P* = 0.032) in Group 3 MB. (**H**) The level of preoperative PLR had no association with OS (*P* = 0.520) in Group 3 MB. (**I**) The level of preoperative NLR exited no statistically significant for PFS (*P* = 0.080) in Group 4 MB. (**J**) High preoperative PLR were associated with poor PFS (*P* = 0.012) in Group 4 MB. (**K**) High preoperative NLR was associated with poor OS (*P* = 0.027) in Group 4 MB. (**L**) High preoperative PLR were associated with poor OS (*P* = 0.009) in Group 4 MB.

There were 23 WNT, 24 SHH, 48 Group 3, and 21 Group 4 MB among 116 MB tumors. When we analysed the survival by restricting the cohort to only these patients, the preoperative NLR in WNT MB were less than 4.94. They were excluded in the survival analysis. It revealed that high preoperative NLR predicted unfavorable OS (*P* = 0.032, Fig. 5G) in Group 3 MB. Similarly, the prognosis of Group 4 MB with high preoperative NLR (OS, *P* = 0.027, Fig. 5K) and PLR (PFS, *P* = 0.012, Fig. 5J, OS, *P* = 0.009, Fig. 5L) were worse than those of Group 4 MB with low NLR and PLR.

## Discussion

Recently, numerous studies have revealed that the preoperative hematological markers played important roles in predicting the prognosis of various tumors^4^. For the first time, we investigated the relationship between preoperative hematological markers and the prognosis of MB patients. We found that high preoperative NLR and PLR predicted unfavorable survival in MB patients, while preoperative MLR, MPV, PDW and AGR had no predictive value on the prognosis in MB patients. In the following multivariate analysis, preoperative NLR and PLR were revealed as independent prognostic factors for the MB patients. Furthermore, we firstly revealed that the levels of preoperative NLR and PLR in Group 3 MB were higher than those in WNT MB. Subsequent survival analysis demonstrated that high preoperative NLR predicted unfavorable OS in Group 3 and Group 4 MB and high preoperative PLR was associated with unfavorable PFS and OS in Group 4 MB.

The exact mechanism about the role of preoperative NLR in the prognosis of tumors need to be fully elucidated. Elevated NLR means more neutrophils, fewer lymphocytes, or both. On the one hand, elevated neutrophils could promote tumor invasion and metastasis by releasing many reactive oxygen species^14^ and cytokines including interleukin-1 (IL-1), tumor necrosis factor (TNF) and vascular endothelial growth factor (VEGF), etc.^15,16^. The reactive oxygen species could induce the DNA damage and genetic instability, and the cytokines promoted to tumor angiogenesis, proliferation and metastasis^17,18^. On the other hand, the tumor infiltrating lymphocytes (TILs) which were differentiated from the T-lymphocytes were considered as systemic and local indicators of anti-tumor reaction. Reduced of TILs were revealed associated with poor prognosis in tumors^19^. A recent study revealed that a decreased infiltrating CD8+ T lymphocyte predicted poor prognosis in patients with MB^20^. Therefore, elevated NLR may indicate the poor prognosis of the patients with MB. In addition, we also found that preoperative NLR in Group 3 MB were significantly higher than those in WNT MB. This finding was in accordance with that the prognosis of WNT MB were significantly better than those in Group 3 MB. In addition, high preoperative NLR was revealed associated with short OS in Group 3 and Group 4 MB. These findings indicated the differential prognostic significances of preoperative NLR in the four molecular subgroups of MB.

The preoperative PLR was also revealed to play an important role in predicting the prognosis in patients with MB. It had been revealed the platelet receptors such as GP1b/IX/V, P-selectin and alphaIIb-beta3 integrin were associated with various tumor progression and metastasis. Additionally, the platelets could release over 30 important angiogenesis regulating proteins in which VEGF was the most important^21^. The levels of VEGF mRNA in Group 3 subgroup (which prognosis is the worst) were significantly higher than those with the other subgroups in patients with MB^22^. Furthermore, the platelets activation and platelets related-protein contributed to the inflammatory response, which could lead to neutrophilia, leukocytosis, thrombocytosis and lymphocytopenia^23,24^. And the platelets protein was reported to enhance the tumor growth and metastasis^21^. Xia, *et al*. and Yun, *et al*. reported that the pretreatment PLR was an independent risk factor for OS in patients with osteosarcoma and renal cell carcinoma^4,24^. In our study, high preoperative PLR was associated with poor PFS and OS in MB patients, particularly high preoperative PLR predicted poor PFS and OS in Group 4 MB.

There exist controversies in the prognostic roles of preoperative MLR, MPV, PDW and AGR in cancers. For example, high preoperative MLR was associated with poor survival in colorectal cancer^3^ and esophageal cancer^2^. However, a recent study revealed that preoperative MLR exhibited no prognostic value in glioblastoma^13^. Similarly, recent studies demonstrated that decreased preoperative MPV had a relationship with unfavorable prognosis of the renal cell carcinoma^9^ and gliomas^25^. On the contrary, a study revealed that high preoperative MPV had poor prognosis in patients with lung cancer^26^. Additionally, Zhang, *et al*. illustrated that decreased preoperative PDW had an unfavorable prognosis in stomach cancer^10^. However, other researches revealed that high preoperative PDW had unfavorable prognosis in laryngeal cancer^27^ and melanoma^28^. Previous studies showed that low preoperative AGR had unfavorable OS in breast cancer and colorectal cancer^29,30^. Our study revealed that preoperative MLR, MPV, PDW and AGR had no prognostic value in MB patients, and the levels of preoperative MLR, MPV, PDW, and AGR among WNT, SHH, Group 3, and Group 4 MB had no significant differences.

Previous study^31^ reported that gross total tumor resection predicted better PFS and OS in MB patients. In our study, the gross total tumor resection correlated with more favorable PFS and OS in our series in the univariate analysis. Moreover, the extent of resection was the independent prognostic factor in MB patients in the multivariate analysis. Therefore, our results corroborated that the extent of resection is one of the most significant predictors of PFS and OS in MB patients.

In the multivariate analyses without molecular subgroups, preoperative NLR and PLR were the independent prognostic factors for PFS and OS in MB patients. When molecular subgroups were added in multivariate analyses, preoperative NLR lost independent significance in the multivariate analysis for PFS. After adding molecular subgroup in the multivariate analyses, preoperative NLR and PLR were still the independent prognostic factors for OS. The OS could be influenced by the differences in the protocols of salvage treatment after tumor recurrence and this might confuse the prognostic significance of hematological markers in MB. Our results indicate preoperative NLR may be influenced by molecular subgroups in PFS. Future cohorts with relatively homogeneous salvage adjuvant therapies and larger sample size are needed to further clarify this question.

In our opinion, we think a One-way ANOVA model with pairwise comparison is appropriate for comparing across multiple groups here. Whereas a One-way ANOVA model assesses whether a significant difference exists among all the groups, pairwise comparisons can be used to determine which groups’ differences are statistically significant. Eckel-Passow, J. E. *et al*.^32^ previously used pairwise comparisons in One-way ANOVA statistics in their paper.

Admittedly, some limitations existed in present study. Firstly, the incidence of MB is low. Some studies reported that the estimated incidence of MB in children was about 0.5/100,000^33,34^. Moreover, MB represented a rare tumor in adults and comprised less than 1% of adult primary brain neoplasms^35^. Compared to previous study^36^, the current cohort of 116 MB patients with complete survival data and molecular subgroup information may not be sufficient to draw final conclusions, especially when the cohort was divided into four molecular subgroups. Therefore, these findings revealed by the current study should be interpreted with caution. Secondly, our research was based on a single-center retrospective study and the findings should be corroborated by multi-centre prospective studies. Thirdly, we revealed preoperative NLR and PLR were correlated with the survival of MB patients based on molecular subgroups, but the exact mechanism between the levels of preoperative NLR, PLR and molecular subgroups need further investigation. Last not the least, according to previous studies^37,38^, patients with disseminated MB are classified according to Chang’s operative staging system. Due to the lack of Chang classification data, we could not investigate whether high preoperative NLR/PLR predict patients with higher probability of experiencing metastasis of MB in the current study.

In conclusion, we firstly demonstrated high preoperative NLR and PLR were significantly correlated with poor survivals of patients with MB. Moreover, the levels of preoperative NLR and PLR in Group 3 MB were significantly higher than those in WNT MB. High preoperative NLR and PLR predict unfavorable survival in Group 3 and Group 4 MB. These findings indicate preoperative NLR and PLR can be used as prognostic predictors for Group 3 and Group 4 MB patients.

## Materials and Methods

### Study population

183 patients were surgically treated in the First Affiliated Hospital of Zhengzhou University from January 2009 to January 2018. The diagnosis of MB was confirmed by postoperative pathology. Patients with hematological diseases, serious infections, severe renal, hepatic dysfunction, diabetes mellitus, metabolic syndrome, surgery, trauma, cardiac-cerebral vascular disease, any therapy with anticoagulant, previous history of infection within 3 months and any inflammatory conditions that could significantly influence preoperative hematological markers were excluded. 39 patients were excluded and 144 patients were enrolled in the cohort. In the 144 MB patients, 116 MB patients were successfully followed up, 28 cases were lost to follow up, (Supplementary Fig. 1). Clinical data including gender, age, preoperative KPS value, tumor location, extent of resection, RT, and CHT were collected from medical records. The follow-up data of the cohort were acquired by telephone follow up or outpatient clinic records. OS was defined as the interval between surgery and death or the end of follow up. PFS was measured from the date of diagnosis to the date of disease recurrence, death, or last follow up.

### Preoperative hematological markers

144 MB patients’ routine blood test and liver function were obtained preoperatively before any treatment. The blood count included blood neutrophil, lymphocyte, platelet, monocyte, MPV, and PDW. The liver function included albumin count, and globulin count. The NLR was equal to the absolute neutrophil count divided by the absolute lymphocyte count, the PLR was equal to the absolute platelet count divided by the absolute lymphocyte count, the MLR was defined as the absolute monocyte count divided by the absolute lymphocyte count, and the AGR was defined as the albumin count divided by globulin count.

### Determination of Molecular subgroups

183 Formalin-fixed paraffin embedded (FFPE) tissues from tumor resection were available in all the cases. RNA was extracted from FFPE tissues, then a nanoString-based assay was employed to test the tumor samples for detection of molecular subgroups according to the Northcott, P. A. *et al*.^39^ previously described.

### Statistical methods

Statistical analyses were performed using SPSS 21.0 (IBM Crop, Armonk, NY, USA), Graph-Pad Prism 5.0 (Graph-Pad Inc, La Jolla, USA) and X-tile 3.6.1 (http://medicine.yale.edu/lab/rimm/research/software.aspx). The differences of preoperative hematological markers between WNT, SHH, Group 3, and Group 4 were compared by One-way ANOVA. Kaplan-Meier method was used to calculate survival curves. The comparison of survival rates in different groups were conducted by the Log-rank test (univariate analysis). Cox proportional hazards regression model was used to evaluate independent prognostic factors (multivariate analysis). Values of *P* < 0.05 were considered as statistically significant.

Our research was approved by the Human Scientific Ethics Committee of the First Affiliated Hospital of Zhengzhou University. All procedures performed in studies involving human participants were in accordance with the ethical standards of the institutional and national research committee and with the 1975 Helsinki declaration and its later amendments or comparable ethical standards. An informed consent was obtained from all individual participants included in the study.

## Supplementary information

Preoperative Neutrophil to Lymphocyte Ratio and Platelet to Lymphocyte Ratio are associated with the prognosis of Group 3 and Group 4 medulloblastoma

## Data Availability

The datasets generated during and/or analyzed during the current study are available from the corresponding author on reasonable request.
