# Supplementary material for: Preoperative Neutrophil to Lymphocyte Ratio and Platelet to Lymphocyte Ratio are Associated with the Prognosis of Group 3 and Group 4 Medulloblastoma
Source: Sci Rep. 2019 Sep 13;9:13239. doi: 10.1038/s41598-019-49733-6 (PMC6744413; doi:10.1038/s41598-019-49733-6)
Supplement: Supplementary file 1 — Preoperative Neutrophil to Lymphocyte Ratio and Platelet to Lymphocyte Ratio are associated with the prognosis of Group 3 and Group 4 medulloblastoma [file 41598_2019_49733_MOESM1_ESM.pdf]

Preoperative Neutrophil to Lymphocyte Ratio and Platelet to Lymphocyte Ratio are associated with the prognosis of Group 3 and Group 4 medulloblastoma

Ke Li[1*], Wen-chao Duan[1*], Hai-biao Zhao[1*], Li Wang[2], Wei-wei Wang[2], Yun-bo Zhan[1], Tao Sun[1], Feng-jiang Zhang[1], Bin Yu[1], Ya-hui Bai[1], Yan-min Wang[1], Yu-chen Ji[1], Jin-qiao Zhou[1], Xian-zhi Liu[1], Zhi-feng Zhang[1], Zhen-yu Zhang[1]

[1]Department of Neurosurgery, The First Affiliated Hospital of Zhengzhou University, Zhengzhou, Henan, China, 450052

[2]Department of Pathology, The First Affiliated Hospital of Zhengzhou University, Zhengzhou, Henan, China, 450052

Corresponding Authors:

Zhen-yu Zhang, M.D. Department of Neurosurgery, First Affiliated Hospital of Zhengzhou University, Jian she Dong Road 1, Zhengzhou, Henan, China, 450052. E-mail: neurozzy@foxmail.com.

Zhi-feng Zhang, M.D. Department of Neurosurgery, First Affiliated Hospital of Zhengzhou University, Jian she Dong Road 1, Zhengzhou, Henan, China, 450052. E-mail: zzfh72@sina.com.

Supplementary Table 1. Preoperative hematological markers are compared with four molecular subgroups by One-way ANOVA. (n = 144).

Parameters	$\bar{x} \pm s$	*P*-value
NLR		
WNT vs. SHH	1.56 ± 0.84 vs. 2.19 ± 1.81	0.529
WNT vs. Group 3	1.56 ± 0.84 vs. 4.02 ± 3.77	**0.007**
WNT vs. Group 4	1.56 ± 0.84 vs. 3.31 ± 5.50	0.070
SHH vs. Group 3	2.19 ± 1.81 vs. 4.02 ± 3.77	**0.034**
SHH vs. Group 4	2.19 ± 1.81 vs. 3.31 ± 5.50	0.223
Group 3 vs. Group 4	4.02 ± 3.77 vs. 3.31 ± 5.50	0.370
PLR		
WNT vs. SHH	110.46 ± 51.28 vs. 130.28 ± 74.98	0.435
WNT vs. Group 3	110.46 ± 51.28 vs. 163.66 ± 74.47	**0.021**
WNT vs. Group 4	110.46 ± 51.28 vs. 167.85 ± 138.77	**0.018**
SHH vs. Group 3	130.28 ± 74.98 vs. 163.66 ± 74.47	0.123
SHH vs. Group 4	130.28 ± 74.98 vs. 167.85 ± 138.77	0.102
Group 3 vs. Group 4	163.66 ± 74.47 vs. 167.85 ± 138.77	0.835
MLR	0.27 ± 0.45	0.544
MPV	8.55 ± 1.42	0.580
PDW	15.68 ± 1.42	0.120
AGR	1.93 ± 0.44	0.675

Supplementary Table 2. Multivariate analysis of prognostic parameters for PFS and OS in medulloblastoma patients (n = 116).

Parameters	PFS		OS	
	OR (95%CI)	*P*-value	OR (95%CI)	*P*-value
Age				
Adults	Reference		Reference	
Children	1.434 (0.513-4.011)	0.492	1.398 (0.393-4.974)	0.605
Extent of resection				
Gross Total	Reference		Reference	
Subtotal	1.900 (0.944-3.824)	0.072	2.543 (1.049-6.166)	**0.039**
RT				
Yes	Reference		Reference	
No	2.930 (1.486-5.777)	**0.002**	4.333 (2.025-9.274)	**<0.001**
Location				
Midline	Reference		Reference	
Lateral	0.735 (0.318-1.699)	0.471	0.726 (0.280-1.833)	0.511
Molecular subgroups				
WNT	Reference		Reference	
SHH	1.478 (0.398-5.487)	0.559	2.338 (0.483-11.312)	0.291
Group 3	3.893 (1.440-10.527)	**0.007**	4.827 (1.383-16.849)	**0.014**
Group 4	3.355 (1.104-10.196)	**0.033**	3.441 (0.859-13.781)	0.081
NLR	1.039 (0.994-1.085)	0.090	1.059 (1.012-1.108)	**0.013**

Supplementary Table 3. Multivariate analysis of prognostic parameters for PFS and OS in medulloblastoma patients (n = 116).

Parameters	PFS		OS	
	OR (95%CI)	*P*-value	OR (95%CI)	*P*-value
Age				
Adults	Reference		Reference	
Children	1.561 (0.555-4.389)	0.399	1.595 (0.442-5.756)	0.476
Extent of resection				
Gross Total	Reference		Reference	
Subtotal	1.892 (0.942-3.800)	0.073	2.520 (1.043-6.089)	**0.040**
RT				
Yes	Reference		Reference	
No	2.988 (1.510-5.913)	**0.002**	4.537 (2.105-9.778)	**<0.001**
Location				
Midline	Reference		Reference	
Lateral	0.682 (0.294-1.582)	0.372	0.638 (0.244-1.665)	0.358
Molecular subgroups				
WNT	Reference		Reference	
SHH	1.522 (0.408-5.683)	0.532	2.491 (0.509-12.199)	0.260
Group 3	4.056 (1.500-10.967)	**0.006**	5.182 (1.480-18.142)	**0.010**
Group 4	3.518 (1.170-10.581)	**0.025**	3.672 (0.929-14.516)	0.064
PLR	1.002 (0.994-1.004)	0.107	1.003 (1.001-1.005)	**0.014**

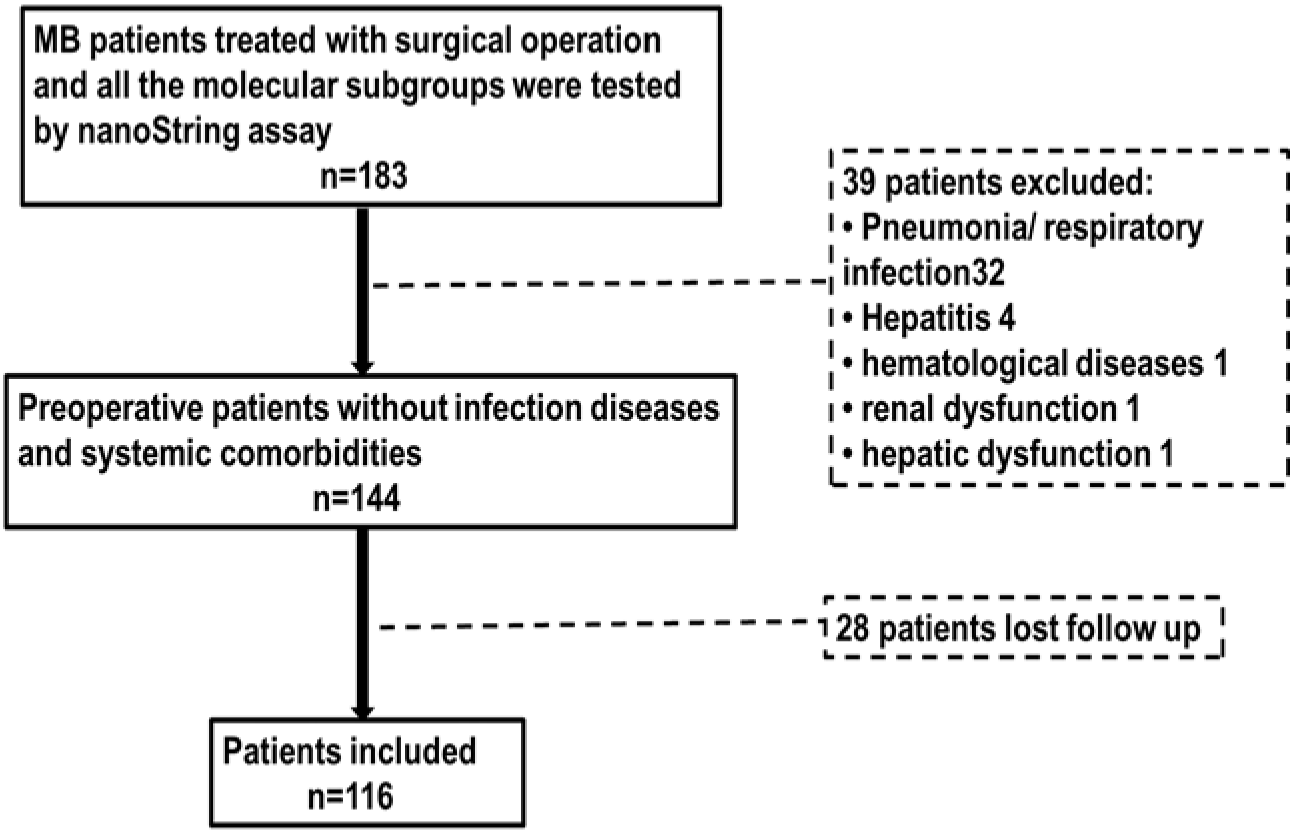

Supplementary Figure 1: Flowchart of patient selection.
